# [Retracted] lncRNA TTN‑AS1 upregulates RUNX1 to enhance glioma progression via sponging miR‑27b‑3p

**DOI:** 10.3892/or.2024.8830

**Published:** 2024-10-21

**Authors:** Keliang Chang, Genwei Wang, Jinfeng Lou, Sha Hao, Ranbo Lv, Desheng Duan, Wanhong Zhang, Yingchang Guo, Pengfei Wang

Oncol Rep 44: 1064–1074, 2020; DOI: 10.3892/or.2020.7684

Following the publication of the above paper, it was drawn to the Editor's attention by a concerned reader that the immunohistochemical images shown in Fig. 7E were strikingly similar to data that had already been published in different form in a previous article in the journal *PLoS One* written by different authors at different research institutes.

Owing to the fact that the abovementioned data had already apparently been published previously, the Editor of *Oncology Reports* has decided that this paper should be retracted from the Journal. The authors were asked for an explanation to account for these concerns, but the Editorial Office did not receive a reply. The Editor apologizes to the readership for any inconvenience caused.

